# Phosphatidylethanol and ethyl glucuronide to categorize alcohol consumption in alcohol-related cirrhosis

**DOI:** 10.1016/j.jhepr.2025.101433

**Published:** 2025-04-24

**Authors:** Benedict T.K. Vanlerberghe, Catalina Dumitrascu, Nele Van den Eede, Hugo Neels, Hannah van Malenstein, Tom J.G. Gevers, Matthijs Kramer, Lukas Van Melkebeke, Ad A.M. Masclee, Douwe de Boer, Schalk van der Merwe, Frederik Nevens, Alexander L.N. van Nuijs, Jef Verbeek

**Affiliations:** 1 Department of Gastroenterology and Hepatology, University Hospitals Leuven, Leuven, Belgium; 2 Laboratory of Hepatology, Department of Chronic Diseases and Metabolism (CHROMETA), KU Leuven, Leuven, Belgium; 3 Department of Gastroenterology and Hepatology, Maastricht University Medical Centre, Maastricht, The Netherlands; 4 School of Nutrition and Translational Research in Metabolism (NUTRIM), Maastricht University, Maastricht, The Netherlands; 5 Department of Pharmaceutical Sciences, Toxicological Centre, University of Antwerp, Antwerp, Belgium; 6 Laboratory Medicine, University Hospitals Leuven, Leuven, Belgium; 7 School for Oncology & Reproduction (GROW), Maastricht University, Maastricht, The Netherlands; 8 Central Diagnostic Laboratory, Maastricht University Medical Centre, Maastricht, The Netherlands

**Keywords:** Alcoholic cirrhosis, Alcoholic liver diseases, Alcohol drinking, Metabolic dysfunction-associated steatotic liver disease, Phosphatidylethanol, Ethyl glucuronide

## Abstract

**Background & Aims:**

Phosphatidylethanol (PEth) is an alcohol-use biomarker that could bridge the detection windows of urinary ethyl glucuronide (uEtG) and scalp hair ethyl glucuronide (hEtG), but has been rarely validated in patients with liver disease. Reported detection windows of these biomarkers also vary significantly, and available studies have focused solely on any alcohol use. Yet, categorizing patients with liver disease based on their level of alcohol use would be highly informative. Here, we assessed the diagnostic accuracy and optimal detection windows of whole-blood PEth, uEtG, hEtG, and the novel biomarker fingernail ethyl glucuronide (nEtG), for different levels of alcohol use in patients with alcohol-related cirrhosis.

**Methods:**

Patients with alcohol-related cirrhosis were questioned on their alcohol use over the previous 3 months using the Alcohol Timeline Followback (n = 116). In addition, 1–7-day (uEtG), 1–5-week (PEth), and 3-month (hEtG and nEtG) detection windows were assessed for any, increased (women ≥2 units/day or men ≥3 units/day), or excessive alcohol use (women ≥5 units/day or men ≥6 units/day).

**Results:**

uEtG, PEth, and hEtG had high diagnostic accuracies for any alcohol use at optimal detection windows of 3 days [area under the receiver operating characteristic curve (AUROC): 0.990 (95% confidence interval (CI): 0.975–1.000)], 3 weeks [AUROC: 0.986 (95% CI: 0.958–1.000)], and 3 months [AUROC: 0.925 (95% CI: 0.862–0.987)], respectively. They had high negative predictive values (>92%) for increased and excessive use. nEtG showed promising results for assessing any alcohol use over the previous 3 months [AUROC: 0.962 (95% CI: 0.924–1.000)].

**Conclusions:**

PEth and EtG have excellent and complementary diagnostic accuracies to detect any alcohol use and rule out increased alcohol use in patients with alcohol-related cirrhosis. nEtG provides an alternative for hEtG, but requires further validation.

**Impact and implications:**

The correct identification and categorization of alcohol use is a major challenge in the treatment of patients with liver disease. Furthermore, given the new nomenclature toward steatotic liver disease, it has become essential to be able to categorize alcohol use into any, increased, or excessive use. The validation of PEth and urine, scalp hair, and nail EtG in patients with alcohol-related liver disease provides us with reliable options to overcome these issues in both clinical care and pharmacological trials on steatotic liver disease.

**Clinical Trials registration:**

The study is registered at ClinicalTrials.gov (NCT04363424).

## Highlights

• uEtG has excellent diagnostic accuracy to detect any alcohol use over the previous 3 days.
• PEth outperforms carbohydrate-deficient transferrin in detecting any alcohol use and, thus, confirming abstinence over the previous 3 weeks.
• hEtG and nEtG demonstrate high diagnostic accuracy in detecting any alcohol use over the previous 3 months.
• Direct alcohol-use biomarkers can reliably exclude increased and excessive alcohol use in patients with ALD.

## Graphical abstract

**Figure:**
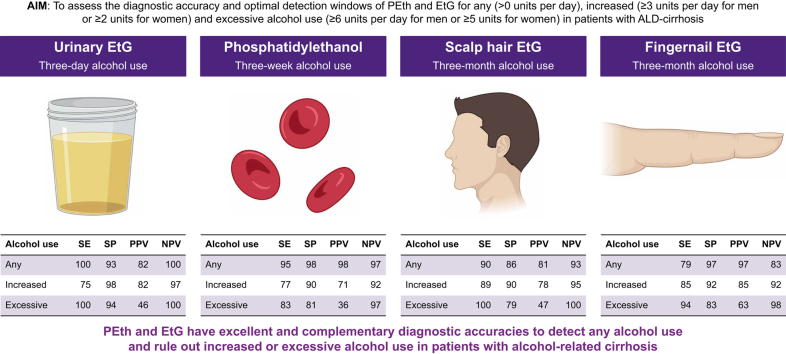

## Introduction

Alcohol-related liver disease (ALD) is the most prevalent cause of cirrhosis and a main indication for liver transplantation.^1^ Abstinence is advised in all patients with liver damage resulting from alcohol.^1^ However, a major challenge in treating patients with ALD is the accurate and objective assessment of abstinence or continued alcohol use.^1^ Patients often deny or under-report their alcohol use,^2^^,^^3^ or can be suspected of alcohol use despite abstaining from alcohol. Therefore, over the past decades, interest has emerged in metabolites of the nonoxidative pathways of ethanol degradation as potential biomarkers of alcohol use.^4^ These direct alcohol-use biomarkers, such as urinary ethyl glucuronide (uEtG), urinary ethyl sulfate (uEtS), EtG in scalp hair (hEtG), and phosphatidylethanol in whole blood (PEth), have been found to be superior over traditional indirect biomarkers, such as liver tests, including gamma-glutamyl transferase or carbohydrate-deficient transferrin (CDT).[4], [5], [6] However, studies of the diagnostic accuracy of direct alcohol-use biomarkers have mainly been performed in forensic contexts and their validation in patients with liver disease is limited, as recently reviewed elsewhere.^4^ Cirrhosis could lead to altered biomarker synthesis, altered hair growth, or decreased biomarker excretion resulting from kidney dysfunction,^7^^,^^8^ which, in turn, could impact alcohol-use biomarker levels. In addition, there is a range of reported detection windows per alcohol-use biomarker, which complicates the interpretation of biomarker results and their implementation.^4^

uEtG and uEtS are short-term biomarkers reflecting alcohol use over the previous days, with reported diagnostic detection windows ranging from 1 to 7 days.^4^ By contrast, hEtG is a long-term alcohol-use biomarker, reflecting alcohol use over the past 3–6 months, depending on the length of analyzed hair.^9^ PEth, which is primarily formed at the membrane of erythrocytes, reflects alcohol use over the previous weeks, yet also shows a high variability in reported diagnostic windows ranging from 2–4 weeks.^4^^,^^10^ Therefore, PEth could fill the gap between the detection windows of uEtG and hEtG, but its diagnostic accuracy in patients with liver disease has only been tested in a few studies.^4^^,^^11^ Fingernail (n)EtG, a novel long-term marker, could provide an alternative for hEtG when hair samples are unavailable. However, to our knowledge, nEtG has not been tested in patients with liver disease.

Apart from diagnosing any alcohol use, alcohol-use biomarkers could also aid the categorization of patients according to their level of alcohol use. This is particularly relevant in patients with presumed metabolic dysfunction-associated liver disease (MASLD) or metabolic dysfunction and alcohol-related liver disease (MetALD),^12^ which, together with ALD, form the spectrum of steatotic liver diseases.^12^ To estimate the contributive effect of alcohol use on the steatotic liver disease phenotype and correct categorization of these patients, insights into absolute levels of alcohol use are required.^12^ However, so far, no studies have systematically assessed the potential of alcohol-use biomarkers to categorize patients with liver disease into increased or excessive alcohol users.^4^

Thus, in this study, we assessed the diagnostic accuracy, potential confounding factors, and optimal detection window of uEtG, uEtS, Peth, and hEtG for the detection of any alcohol use in patients with ALD cirrhosis and their potential to categorize patients according to their level of alcohol use. In addition, we assessed, for the first time, the utility of nEtG in these patients.

## Methods

### Study population

Patients were included who attended the in- and outpatient clinics of University Hospitals Leuven and the Maastricht University Medical Center between July 1, 2020 and June 1, 2022. Adult patients (age ≥18 years) with ALD cirrhosis who could recall their alcohol use in the previous 3 months were eligible for inclusion. The diagnosis of ALD was based on patient history and the exclusion of other liver diseases (viral hepatitis, hereditary hemochromatosis, Wilson’s disease, autoimmune hepatitis, primary biliary cholangitis, and primary sclerosing cholangitis). The presence of cirrhosis was determined by imaging (ultrasound, computed tomography, or magnetic resonance imaging) and/or histological examination of the liver. After visiting the treating physician, patients willing to participate in the study were seen independently by the research physician. As a next screening step before inclusion, alcohol use on a day-by-day basis over the previous 3 months was assessed by the research physician by applying the Alcohol Timeline Followback Method. In patients who did not report consuming any alcohol over the previous 90 days, the date of their last alcohol use was recorded. Patients were included in cases where there was no discrepancy between alcohol use reported to the treating physician and to the research physician, and if there was a trustworthy recall of alcohol use over the previous 3 months.

After inclusion, patients who denied any alcohol use over the previous 3 months with at least two positive results out of three biomarkers (uEtG, PEth, or hEtG) were excluded. Patient characteristics were collected from the medical record. Information on hair coloring, nail polish or detergent use, use of alcohol-based hand sanitizer, symptoms suggestive of urinary tract infection, and medication use was recorded.

The study was approved by the ethical committees of the University Hospitals Leuven (S63594) and the Maastricht University Medical Center (NL71593.068.19). It was conducted in accordance with the Declaration of Helsinki (2013) and the Standards for Reporting of Diagnostic Accuracy Studies (STARD).^13^ Written informed consent was obtained from each patient.

#### Definitions

Any alcohol use was defined as having any alcoholic beverage during the reported detection window.^12^ Increased alcohol use was defined as drinking three or more units per day for men and two or more units per day for women on average during the reported detection window.^12^ Excessive alcohol use was defined as consuming six or more units per day for men and five or more units per day for women on average during the reported detection window.^12^ A standard unit of alcohol was defined as containing 10 g of alcohol.^1^ Diagnostic accuracy results that have not been adjusted for alcohol use based on sex (for both women and men: three or more units per day for increased and six or more units per day for excessive use) can be found in Table S1). Diabetes mellitus was defined as having, or receiving treatment for, type 2 diabetes mellitus. Arterial hypertension was defined as having a blood pressure ≥130/85 mmHg or receiving specific antihypertensive drug treatment. Hypercholesterolemia was defined as having an HDL cholesterol ≤1.0 mmol/L for men or ≤1.3 mmol/L for women or receiving lipid-lowering treatment, and hypertriglyceridemia as having triglycerides ≥1.7 mmol/L or receiving lipid-lowering treatment.

### Alcohol-use biomarker analysis

After study inclusion, blood, urinary, fingernail, and hair samples were collected during the study visit. Urine samples were tested for uEtG and uEtS by liquid chromatography–tandem mass spectrometry (LC-MS/MS). The applied cut-off value for any alcohol use was ≥0.121 mg/g creatinine for uEtG (based on a uEtG cut-off of ≥100 mg/dl^14^) and ≥ 0.108 mg/g creatinine for uEtS. Patients with urinary creatinine <20 mg/dl were excluded from analysis because samples were too diluted for analysis. Furthermore, 1- to 7-day windows were analyzed to determine the optimal detection window. A cut-off of ≥9 mg/g creatinine uEtG was used to detect increased or excessive alcohol use and was based on the upper limit of quantification of commercial uEtG detection kits (9.84 mg/L uEtG). The diagnostic accuracy of uEtG and uEtS not corrected for urinary creatinine is detailed in Table S2.

PEth 16.0/18:1 was analyzed by LC-MS/MS as described elsewhere.^15^ The cut-off for any alcohol use was PEth ≥20 ng/ml,^16^ and the cut-off for increased or excessive drinking was set at ≥200 ng/ml. The upper limit of quantification for analysis of PEth 16:0/18:1 was 2,000 ng/ml.^15^ Detection windows ranging from 1 to 5 weeks were compared for PEth. CDT was tested by both nephelometry [CDT_(Nef)_] and capillary zone electrophoresis [CDT_(CZE)_], with a cut-off for abstinence, increased, and excessive alcohol use, defined by the International Federation of Clinical Chemistry and Laboratory Medicine (IFCC) of 1.7% CDT_IFCC_.^17^ Patients with atypical profiles suggesting 2-3-sialo block interference (di-trisialotransferrin bridging) in capillary zone electrophoresis (CZE) were excluded.^18^

EtG testing in hair was performed by gas chromatography-tandem mass spectrometry (GC-MS/MS) as previously described.^9^^,^^15^ The proximal 3-cm vortex scalp hair strand was analyzed. The applied cut-off for any alcohol use in hair samples was ≥5 pg/mg and ≥30 pg/mg for increased or excessive alcohol use over the previous 3 months.^19^ EtG testing in nails was performed by GC-MS/MS,^20^ with a cut-off of ≥59 pg/mg nail for any and ≥123 pg/mg nail for increased or excessive alcohol use over the previous 3 months.^20^^,^^21^ Nail samples were collected by clipping of the distal edges of all ten fingernails.^20^ Both nail and hair samples were stored at room temperature in aluminum foil until analysis.^20^ Both hEtG and nEtG had an upper quantification limit of 300 pg/mg. No adverse events occurred during sample collection.

Other indirect biomarkers were analyzed with the following cut-offs for any, increased, and excessive use: mean corpuscular volume (MCV), >96 fl; gamma-glutamyl transferase (GGT), >60 U/L for men and >40 U/L for women; aspartate transferase (AST), >37 U/L for men and >31 U/L for women; alanine transaminase (ALT), >41 U/L for men and >31 U/L for women. The diagnostic accuracy of these markers can be found in Table S3. The reference test results were not available to those performing the biomarker analysis.

### Statistical analysis

For each alcohol-use biomarker, discriminative power was evaluated by area under the receiver operating characteristic curve (AUROC), sensitivity, specificity, positive predictive value (PPV), negative predictive value (NPV), and Youden index. The range of the absolute levels of each biomarker is presented by box plots (showing IQRs and minimal and maximal values). Tests with an AUROC of 0.97–1.00 are considered to have excellent discriminative properties, with a value of 0.93–0.96 as very good and of 0.75–0.92 as good.^22^ Tests with an AUROC <0.75 are not considered to have good clinical discriminative properties.^22^ AUROCs are presented for all biomarkers and all figures were drawn using GraphPad PRISM version 10.0. Predefined cut-offs for alcohol-use biomarkers (based on literature^4^^,^^17^) were used to assess diagnostic accuracy, which were then compared with the optimal detection cut-off of each biomarker. The optimal cut-offs were calculated using the value corresponding with the highest Youden index based on the AUROC.

Normality was checked by the Shapiro–Wilk test. Qualitative variables were compared using the χ^2^ test. Normally distributed values are presented as means with 95% CI or SD and were compared using an independent t-test. Non-normally distributed values are presented as medians with IQR and were compared using the Mann-Whitney *U* and Kruskal–Wallis tests. Statistical significance was set at *p* ≤0.05. Pairwise deletion was used to handle missing data. To analyze the association between absolute values of alcohol-use biomarkers and covariates, univariate and multivariate linear regressions were performed. Covariates were chosen based on previous literature and expert opinion. Child-Pugh scores were assessed by group (A *vs.* B *vs.* C), and kidney dysfunction was defined as non-Kidney Disease Improving Global Outcomes (KDIGO) stage 1 [estimated glomerular filtration rate (eGFR) <90 ml/min/1.73 m^2^]. Absolute values of alcohol-use biomarkers were transformed (squared transformation) to fit normality assumptions. All statistical analyses were performed using SPSS version 29.0.

## Results

### Patient characteristics

In total, 116 patients were included in the study, 109 at the University Hospitals Leuven and seven at the Maastricht University Medical Center. Six patients with self-reported abstinence in the previous 3 months were excluded because of two positive biomarker results (Fig. S1). Therefore, 110 patients were included in the analysis (Table 1). Any alcohol use over the previous 3 months was reported by 48 (43.6%) patients and abstinence over the previous 3 months was reported by 62 (56.4%) patients (Table 1). Patients were mostly men (n = 82; 74.5%) and their mean age was 60.7 years (SD: 10.8). Patients with any alcohol use over the previous 3 months were significantly younger than those without any alcohol use (*p* = 0.037). Out of the 110 included patients, 66 (60.0%) had Child-Pugh A cirrhosis, 34 (30.9)%) had Child-Pugh B cirrhosis, and 10 (9.1%) had Child-Pugh C cirrhosis. Child-Pugh scores did not differ significantly between patients with and without any alcohol intake over the previous 3 months compared with abstainers (Table 1). Mean BMI and the rate of other dysmetabolic features (diabetes mellitus type 2, arterial hypertension, hypercholesterolemia, and hypertriglyceridemia) are detailed in Table 1.

**Table 1:** Patient characteristics.

Patient characteristics	Overall (n = 110)	Any alcohol use over previous 3 months (n = 48)	Abstinent over previous 3 months (n = 62)	*p* value
Age (years)	60.7 (10.7)	58.2 (11.7)	62.5 (9.7)	**0.037∗**
Male	82 (74.5)	36 (75)	46 (74.2)	0.923
BMI	27.7 (4.9)	27.5 (5.3)	27.8 (4.6)	0.745
Child-Pugh score	6.0 (5.0–8.0)	6.0 (5.0–9.0)	5.0 (5.0–7.3)	0.136
Child-Pugh A	66 (60.0)	25 (52.1)	41 (64.5)	0.136
Child-Pugh B	34 (30.9)	19 (39.6)	15 (24.2)	0.083
Child-Pugh C	10 (9.1)	4 (8.3)	6 (11.3)	0.808
Lab MELD score	10.0 (7.75–16.0)	12.5 (7.25–18.0)	9.0 (7.75–16.0)	0.581
Bilirubin (mg/dl)	1.02 (0.60–2.41)	1.35 (0.73–4.54)	0.89 (0.52–1.59)	**0.007∗**
AST (U/L)	38.0 (27.0–62.3)	54.5 (32.3–123.8)	32.0 (25.0–44.8)	**0.002∗**
GGT (U/L)	98.0 (40.8–223.8)	218.0 (100.5–465.5)	58.5 (33.0–113.8)	**<0.001∗**
INR	1.23 (1.10–1.50)	1.25 (1.10–1.61)	1.23 (1.10–1.50)	1.000
Hemoglobin (g/dl)	13.1 (10.8–15.2)	13.1 (11.3–14.9)	13.4 (10.5–15.4)	0.473
Creatinine (mg/dl)	0.84 (0.65–1.01)	0.74 (0.60–0.99)	0.90 (0.73–1.08)	0.060
KDIGO 1	63 (57.3)	32	31	0.079
KDIGO 2	31 (28.2)	12	19	0.514
KDIGO 3	11 (10.0)	2	9	0.073
KDIGO 4	4 (3.6)	1	3	0.444
KDIGO 5	1 (0.9)	1	0	N.A.
Loop diuretic use	35 (31.8)	11 (22.9)	24 (38.7)	0.078
Mineralocorticoid antagonist use	45 (40.1)	15 (31.3)	30 (48.4)	0.070
Type 2 diabetes mellitus	25 (22.7)	7 (14.6)	18 (29.0)	0.073
Arterial hypertension	33 (30.0)	13 (27.1)	20 (32.3)	0.661
Hypercholesterolemia	32 (29.1)	15 (31.3)	17.4 (27.4)	0.557
Hypertriglyceridemia	33 (30.0)	15 (31.3)	18 (29.0)	0.801
AUDIT score	5.5 (2.8–17.0)	17.0 (7.3–29.0)	3.5 (2.0–4.0)	**<0.001∗**
Hair coloring	13 (11.8)	5 (10.4)	8 (12.9)	0.689
Use of nail polish/treatment	13 (11.8)	7 (14.6)	6 (9.7)	0.429
Daily use of alcohol-based hand sanitizer	84 (76.4)	36 (75.0)	48 (77.4)	0.767

**Alcohol use**				
Previous 3 days: any use	27 (24.5)	27 (56.3)	0 (0)	N.A.
Previous 3 days: increased use	15 (13.6)	15 (31.3)	0 (0)	N.A.
Previous 3 days: excessive use	8 (7.3)	8 (16.7)	0 (0)	N.A.
Previous 3 weeks: any use	43 (39.1)	43 (89.6)	0 (0)	N.A.
Previous 3 weeks: increased use	26 (23.6)	26 (54.2)	0 (0)	N.A.
Previous 3 weeks: excessive use	12 (10.9)	12 (25.0)	0 (0)	N.A.
Previous 3 months: any use	48 (43.6)	48 (100)	0 (0)	N.A.
Previous 3 months: increased use	32 (29.1)	32 (66.7)	0 (0)	N.A.
Previous 3 months: excessive use	19 (17.3)	19 (39.6)	0 (0)	N.A.

Data are reported as the number of patients with percentage, mean with SD or median with IQR as appropriate. Comparisons were made using the χ2 test, independent t-test, or Mann-Whitney *U* test as appropriate. Statistical significance was set at *p* ≤0.05 (denoted by ∗). AST, aspartate transferase; AUDIT, alcohol use disorder identification test; GGT, gamma-glutamyl transferase; INR, international normalized ratio; KDIGO, Kidney Disease Improving Global Outcomes; MELD, model for end-stage liver disease.

### Diagnostic accuracies for any alcohol use

#### Short-term alcohol use biomarkers (uEtG and uEtS)

**Fig. 1:**
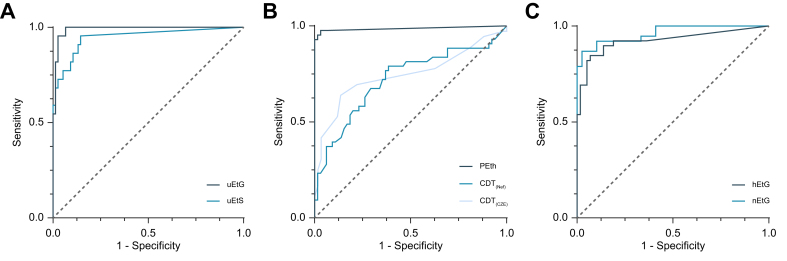
Direct alcohol-use biomarkers to detect any alcohol use. ROCs of (A) uEtG and uEtS for any alcohol use at a 3-day detection window, (B) PEth, CDT_(Nef)_, and CDT_(CZE)_ for any alcohol use at a 3-week detection window, and (C) of hEtG and nEtG for any alcohol use at a 3-month detection window. CDT_(Nef)_, carbohydrate-deficient transferrin by nephelometric analysis; hEtG, hair ethyl glucuronide; nEtG, nail ethyl glucuronide; ROC, receiver operating characteristic curve; uEtG, urinary ethyl glucuronide.

**Fig. 2:**
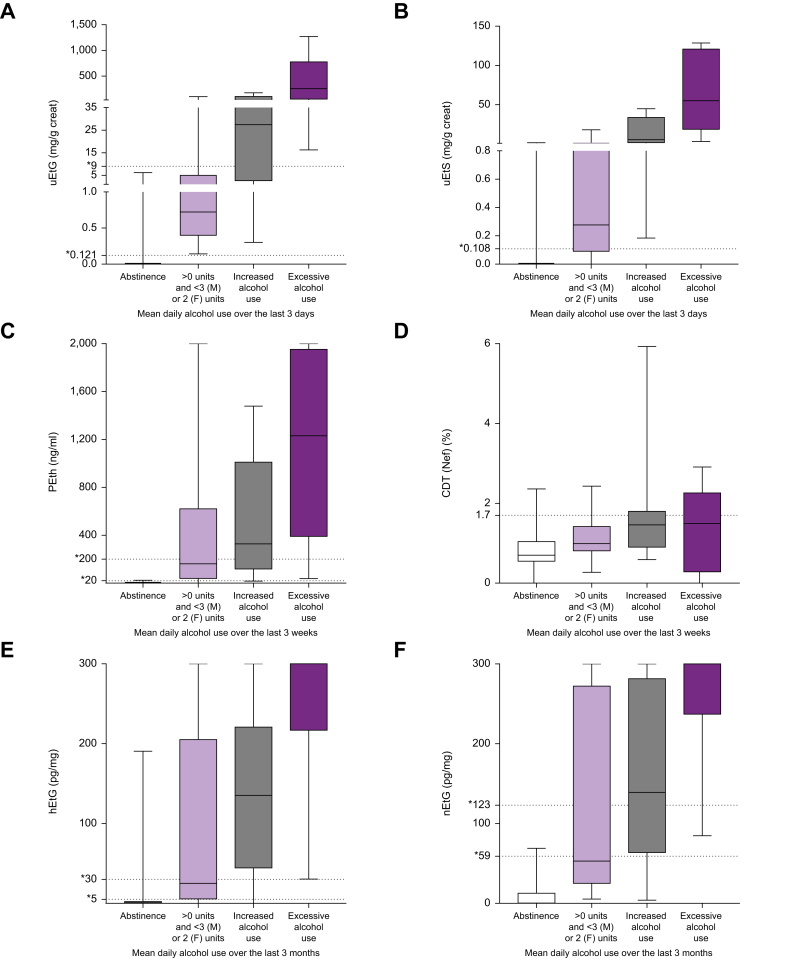
Absolute levels of direct alcohol-use biomarkers per alcohol use category. Box plots representing absolute levels of (A) uEtG, (B) uEtS, (C) PEth, (D) CDT_(Nef)_, (E) hEtG, and (F) nEtG per alcohol use category. Bars represent IQR, bold lines inside the box plot indicate median levels, and whiskers represent minimum and maximum values. CDT_(Nef)_: carbohydrate-deficient transferrin by nephelometric analysis; hEtG, hair ethyl glucuronide; nEtG, nail ethyl glucuronide; PEth, phosphatidylethanol; uEtG, urinary ethyl glucuronide; uEtS, urinary ethyl sulfate.

Overall, 100 patients (90.9%) provided a urine sample for uEtG and uEtS analysis. Two patients were excluded because their urine was too dilute for analysis. The diagnostic accuracy of uEtG for any alcohol use was the highest when analyzing alcohol use over the previous 3 days (Table S4), with an AUROC of 0.990 (9%5 CI: 0.975–1.004) and a Youden index of 0.94 (Table 2; Fig. 1A). Absolute values of uEtG ranged from 0.0 to 6.188 mg/g creatinine in those without any alcohol use over the previous 3 days (median 0.0; IQR: 0.0–0.0) and 0.142 to 1,270.8 mg/g creatine in those with alcohol use over the previous 3 days (median: 10.57; IQR: 0.55 –102.6) (Fig. 2A). At every analyzed diagnostic time window (1, 2, 3, 4, 5, 6, and 7 days), uEtG showed a higher AUROC and Youden index compared with uEtS (Table S4). Patients with a discrepant uEtG value over a 3-day detection window (*i.e.* positive value despite reporting a 3-day abstinence) last used alcohol at day 7 before inclusion (n = 2) or between 14 and 18 days before inclusion (n = 3).

**Table 2:** Diagnostic accuracy of uEtG, uEtS, PEth, CDT_(Nef)_, CDT_(CZE)_, hEtG, and nEtG to detect any, increased or excessive alcohol use.

Alcohol-use biomarker	AUROC (95% CI)	Sensitivity (%)	Specificity (%)	PPV (%)	NPV (%)	Youden index
**Short-term alcohol-use biomarkers**						
Short-term alcohol-use biomarkers reflecting 3-day any alcohol use						
uEtG (≥0.121 mg/g creat)	0.990 (0.975–1.000)	100	93	82	100	0.934
uEtS (≥0.108 mg/g creat)	0.948 (0.889–1.000)	86	88	68	96	0.746
Short-term alcohol-use biomarkers reflecting 3-day increased alcohol use						
uEtG (≥9 mg/g creat)	0.982 (0.957–1.000)	75	98	82	97	0.727
Short-term alcohol-use biomarkers reflecting 3-day excessive alcohol use						
uEtG (≥9 mg/g creat)	0.983 (0.956–1.000)	100	94	46	100	0.935

**Mid-term alcohol-use biomarkers**						
Mid-term alcohol-use biomarkers reflecting 3-week any alcohol use						
PEth (≥20 ng/ml)	0.986 (0.958–1.000)	95	98	98	97	0.936
CDT_(Nef)_ (>1.7%)	0.721 (0.618–0.825)	30	94	77	67	0.240
CDT_(CZE)_ (>1.7%)	0.744 (0.630–0.859)	31	97	85	70	0.272
Mid-term alcohol-use biomarkers reflecting 3-week increased alcohol use						
PEth (≥200 ng/ml)	0.936 (0.892–0.981)	77	90	71	92	0.668
CDT_(Nef)_ (>1.7%)	0.716 (0.586–0.845)	39	92	59	82	0.300
CDT_(CZE)_ (>1.7%)	0.779 (0.642–0.915)	40	93	62	85	0.333
Mid-term alcohol use biomarkers reflecting 3-week excessive alcohol use						
PEth (≥200 ng/ml)	0.921 (0.861–0.980)	83	81	36	97	0.639
CDT_(Nef)_ (>1.7%)	0.665 (0.445–0.885)	50	89	35	93	0.385
CDT_(CZE)_ (>1.7%)	0.745 (0.540–0.949)	33	88	23	93	0.217

**Long-term alcohol-use biomarkers**						
Long-term alcohol-use biomarkers reflecting 3-month any alcohol use						
hEtG (≥5 pg/mg)	0.925 (0.862–0.987)	90	86	81	93	0.759
nEtG (≥59 pg/mg)	0.962 (0.924–1.000)	79	97	97	83	0.763
Long-term alcohol-use biomarkers reflecting 3-month increased alcohol use						
hEtG (≥30 pg/mg)	0.906 (0.829–0.983)	89	90	78	95	0.792
nEtG (≥123 pg/mg)	0.941 (0.886–0.995)	85	92	85	92	0.772
Long-term alcohol use biomarkers reflecting 3-month excessive alcohol use						
hEtG (≥30 pg/mg)	0.942 (0.895–0.989)	100	79	47	100	0.793
nEtG (≥123 pg/mg)	0.946 (0.889–0.994)	94	83	63	98	0.775

Data are reported as AUROC and 95% CI, sensitivity, specificity, PPV, NPV, and Youden index as appropriate. AUROC, area under the receiver operating characteristic curve; CDT_(CZE)_, carbohydrate-deficient by capillary zone electrophoresis; CDT_(Nef)_, carbohydrate-deficient transferrin by nephelometric analysis; creat, creatinine; hEtG, scalp hair ethyl glucuronide; nEtG, fingernail ethyl glucuronide; NPV, negative predictive value; PEth, phosphatidylethanol; PPV, positive predictive value; uEtG, urinary ethyl glucuronide; uEtS, urinary ethyl sulfate.

#### Intermediate-term alcohol use biomarkers (PEth and CDT)

In total, 108 patients delivered blood samples, of which 105 could be analyzed for PEth, 108 for CDT_(Nef)_, and 95 for CDT_(CZE)_. The diagnostic accuracy of PEth for any alcohol use was highest at a 3-week detection window (Table S5), corresponding with an AUROC of 0.986 (95% CI: 0.958–1.000) and a Youden index of 0.936 (Table 2; Fig. 1B). Absolute values of PEth ranged from 0.0 to 23.4 ng/ml in those without any alcohol use over the previous 3 weeks (median 0.0; IQR: 0.0–0.0)) and from 0.0 to 2,000.0 ng/ml in those with alcohol use over the previous 3 weeks (median: 362.8; IQR: 83.4–1,211.9) (Fig. 2C). CDT showed lower diagnostic accuracy at every analyzed time window compared with Peth, with AUROCs <0.750 and Youden indexes of 0.240 for CDT_(Nef)_ and 0.272 for CDT_(CZE)_ using the 3-week detection window (Table S5).

Two out of 105 patients who underwent a PEth test tested false negative and one patient had a discrepant positive test result. The two patients with a false negative result had consumed alcohol up to 9 and 15 days before inclusion, respectively. The first had an average intake of 0.1 units a day over the previous 21 days with an undetectable PEth and the second patient had a mean alcohol intake of 4.66 units a day with a PEth of 16.7 ng/ml. The discrepant positive PEth result (*i.e.* positive value despite reporting a 3-week abstinence) occurred in a patient who reported alcohol use until 32 days before inclusion, with a slightly elevated PEth of 23.4 ng/ml; he had drunk heavily between 33 and 90 days before inclusion (mean intake 4.51 units a day over the previous 3 months) (Table 2).

#### Long-term alcohol use biomarkers (hEtG and nEtG)

Out of 97 patients (88.2%) who provided hair samples, 39 (40.2%) reported any alcohol use over the previous 3 months, resulting in an AUROC of 0.925 (95% CI: 0.832–0.987) and a Youden index of 0.759 (Table 2) (Fig. 1C). Absolute values of hEtG ranged from 0.0 to 190.6 pg/mg in those without any alcohol use over the previous 3 months (median 0.0; IQR: 0.0–1.8)) and from 0.0 to 300.0 pg/mg in those with alcohol use over the previous 3 months (median: 200.0; IQR: 29.0–300.0) (Fig. 2E). Four patients had a false negative result (*i.e.* <5 pg/mg), with a self-reported last day of alcohol use 1, 9, 32, and 64 days before inclusion, respectively. They had an average 3-month alcohol intake of 2.62 units/day, 0.13 units/day, 4.51 units/day, and 5.77 units/day before inclusion, respectively. In these patients with false negative hEtG, PEth was positive in the patients with a self-reported last day of alcohol use 1 and 32 days before inclusion and was negative in the other two. Eight patients had a discrepant positive hEtG result, of which four had a self-reported last day of alcohol use 120 days (hEtG 50.7 pg/mg), 130 days (hEtG 190.6 pg/mg), 150 days (hEtG: 9.0 pg/mg), and 160 days (6.1 pg/mg) before inclusion, respectively. The other patients with a positive result despite reported abstinence, reported a last day of alcohol use more than 500 days before inclusion (hEtG levels of 6.2, 8.1, 19.4, and 68.0 pg/mg, respectively). Out of the eight patients with a discrepant positive hEtG, six had a known PEth, which was negative.

Seventy-seven patients (70%) provided fingernail samples. nEtG had a higher AUROC of 0.962 (95% CI: 0.924–1.000) for detecting any alcohol use during the previous 3 months compared with during the previous 3 weeks (0.938; 95% CI: 0.885–0.992) (Table 2; Fig. 1C). Absolute values of nEtG ranged from 0.0 to 68.94 pg/mg in those without any alcohol use over the previous 3 months (median 0.3; IQR: 0.0–12.7)) and from 3.9 to 300.0 pg/mg in those with alcohol use over the previous 3 months (median: 254.7; IQR: 83.7–300.0) (Fig. 2F). Eight patients had a false negative nEtG test. They had a self-reported last day of alcohol use ranging from 1 day to 32 days before inclusion. Out of the eight patients with a false negative nEtG, PEth and hEtG samples were available for seven patients, with six patients testing positive for PEth and four for hEtG. One patient had a positive result despite reported abstinence, with a nEtG of 68.9 pg/mg. His self-reported last day of alcohol use was 150 days before inclusion and he had a negative PEth and a positive hEtG test (9.0 pg/mg).

#### Diagnostic accuracy for increased and excessive alcohol use

**Fig. 3:**
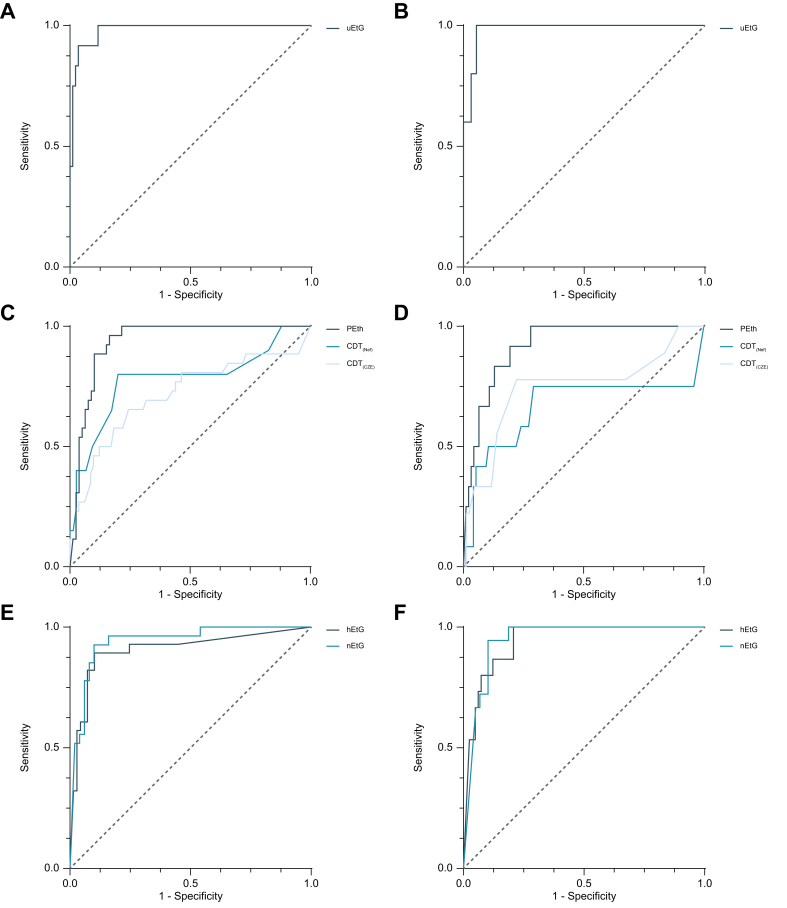
Direct alcohol-use biomarkers to detect increased and excessive alcohol use. ROCs of uEtG for (A) increased alcohol use and (B) excessive alcohol use at a 3-day detection window. ROCs of PEth, CDT_(Nef)_ and CDT_(CZE)_ for (C) increased alcohol use and (D) excessive alcohol use at a 3-week detection window. ROCs of hEtG and nEtG for (E) increased alcohol use and (F) excessive alcohol use at a 3-month detection window. CDT_(CZE)_: carbohydrate-deficient by capillary zone electrophoresis; CDT_(Nef)_, carbohydrate-deficient transferrin by nephelometric analysis; hEtG, hair ethyl glucuronide; nEtG, nail ethyl glucuronide; PEth, phosphatidylethanol; ROC, receiver operating characteristic curve; uEtG, urinary ethyl glucuronide;

uEtG had an AUROC of 0.982 (95% CI: 0.957–1.000) and a Youden index of 0.727 to detect increased alcohol use over the previous 3 days using a cut-off of 9 mg/g creatinine. Applying this cut-off to analyze excessive alcohol use resulted in a Youden index of 0.935 with an AUROC of 0.983 (95% CI: 0.953–1.000) (Table 2; Fig. 3).

PEth had an AUROC of 0.936 (95% CI: 0.892–0.981) and a Youden index of 0.668 to detect increased alcohol use over the previous 3 weeks using a cut-off of ≥200 ng/ml. Applying a ≥200 ng/ml cut-off to analyze excessive alcohol use resulted in a Youden index of 0.639 with an AUROC of 0.921 (95% CI: 0.861–0.980) (Table 2; Fig. 3).

hEtG had an AUROC of 0.906 (95% CI: 0.829–0.983) and a Youden index of 0.792 (Table 2) to detect increased alcohol use (hEtG ≥30 pg/mg) over the previous 3 months. Using the same cut-off for excessive use resulted in a Youden index of 0.793 with an AUROC of 0.942 (95% CI: 0.895–0.989) to detect excessive alcohol use over the previous 3 months (Table 2; Fig. 3).

nEtG had an AUROC of 0.941 (95% CI: 0.886–0.995) and a Youden index of 0.773 to detect increased alcohol use (nEtG ≥123 pg/mg). When applying the same cut-off for excessive use, this resulted in an AUROC of 0.946 (95% CI: 0.889–0.994) and a Youden index of 0.775 (Table 2: Fig. 3).

#### Optimal detection window

We found only small differences when comparing the predefined cut-offs based on published literature to the optimal detection cut-off based on the highest Youden index. This resulted in only a limited number of patients who would have been categorized differently when using the optimal cut-off compared with the predefined cut-off (Table S6).

### Impact of liver and kidney function and diuretics use

In univariate analysis, absolute uEtG levels correlated with the level of alcohol use over the previous 3 days (B (unstandardized coefficient): 0.860; SD: 0.042; *p* <0.001), but not with Child-Pugh score (group comparison), female sex, kidney dysfunction (defined as non-KDIGO 1), BMI, sex, or loop diuretics use (Table 3). On multivariate analysis, both alcohol use over the previous 3 days (B: 0.861; SD: 0.041; *p* <0.001) and loop diuretics use (B: -0.982; SE: 0.471; *p* = 0.040) showed a significant correlation with absolute uEtG levels. There was no difference in discrepant positive uEtG results in those taking loop diuretics compared with those not on diuretics (4.5% *vs.* 6.3%, *p* = 0.720).

**Table 3:** Uni- and multivariate linear regression analysis analyzing the correlation between absolute levels of alcohol use biomarkers (after square root transformation) and patient characteristics.

	Univariate				Multivariate			
Characteristic	B	SE	Beta	*p* value	B	SE	Beta	*p* value
**Urinary EtG**								
Alcohol use∗	0.860	0.042	0.903	**<0.001**	0.861	0.041	0.904	**<0.001**
Child-Pugh score	-0.134	0.749	-0.018	0.859	0.530	0.353	0.072	0.137
Kidney dysfunction†	-0.397	0.984	-0.041	0.688	0.778	0.435	0.081	0.077
Female sex	0.427	1.086	0.040	0.695	0.805	0.463	0.076	0.086
BMI (kg/m^2^)	0.053	0.100	0.054	0.596	0.041	0.046	0.042	0.376
Loop diuretics use	-1.697	1.009	-0.169	0.096	-0.982	0.471	-0.098	**0.040**

**Phosphatidylethanol**								
Alcohol use∗	1.594	0.191	0.636	**<0.001**	1.466	0.196	0.578	**<0.001**
Child-Pugh score	4.687	2.015	0.223	**0.022**	4.834	1.857	0.233	**0.011**
Kidney dysfunction†	-7.162	2.682	-0.255	**0.009**	-2.769	2.231	–0.099	0.218
Female sex	0.911	3.178	0.028	0.775	1.871	2.490	0.059	0.454
BMI (kg/m^2^)	-0.009	0.282	-0.003	0.975	0.155	0.233	0.055	0.508
Hemoglobin	0.254	0.500	0.050	0.507	0.875	0.477	0.173	0.069

**Scalp hair EtG**								
Alcohol use∗	0.931	0.097	0.703	**<0.001**	0.920	0.105	0.695	**<0.001**
Child-Pugh score	2.108	0.981	0.215	**0.034**	0.072	0.787	0.007	0.928
Kidney dysfunction†	-2.516	1.353	-0.187	0.066	-0.167	1.041	-0.012	0.873
Female sex	0.044	1.513	0.003	0.977	0.225	1.109	0.015	0.840
BMI (kg/m^2^)	-0.165	0.141	-0.119	0.0247	-0.093	0.110	-0.067	0.403

**Fingernail EtG**								
Alcohol use∗	0.862	0.088	0.747	**<0.001**	0.844	0.095	0.731	**<0.001**
Child-Pugh score	2.974	1.089	0.282	**0.012**	1.187	0.820	0.120	0.152
Kidney dysfunction†	-2.829	1.563	-0.202	0.074	0.258	1.128	0.019	0.820
Female sex	1.029	1.794	0.066	0.568	1.179	1.221	0.075	0.337
BMI (kg/m^2^)	-0.146	0.152	-0.110	0.338	0.054	0.109	0.040	0.622

Correlation between absolute levels of alcohol-use biomarkers (after square root transformation) and patient characteristics were assessed by univariate and multivariate linear regression. Statistical significance was set at *p* ≤0.05 (in bold).

B, unstandardized coefficient ; EtG: ethyl glucuronide; KDIGO, Kidney Disease Improving Global Outcomes.

∗ Alcohol use defined as mean units of alcohol a day over the previous: 3 days for uEtG, 3 weeks for PEth, and 3 months for hEtG and nEtG before inclusion.

† kidney dysfunction defined as non-KDIGO 1.

PEth correlated with alcohol use over the previous 3 weeks (B: 1.594; SD: 0.191; *p* <0.001), Child-Pugh score (B: 4.687; SD: 2.015; p = 0.022) and kidney dysfunction (B: -7.162; SD: 2.682); *p* = 0.009) and not with hemoglobin levels or female sex on univariate analysis. On multivariate analysis, only alcohol use over the previous 3 weeks (B: 1.466; SD: 0.196; *p* <0.001) and Child-Pugh score (B: 4.834; SD: 1.857; *p* = 0.011) remained statistically significant (Table 3). There was no difference in either discrepant positive results (0% *vs.* 2.3%, *p* = 0.227) or false negative PEth results (1.6% *vs.* 2.3%, *p* = 793) in patients with Child-Pugh A compared with patients with Child-Pugh B or C.

hEtG was associated with alcohol use over the previous 3 months (B: 0.931; SD: 0.097; *p* <0.001) and Child-Pugh score (B: 2.108; SD: 0.981; *p* = 0.034) in univariate linear regression; after multivariate regression, only alcohol use remained statistically significant. nEtG correlated with alcohol use over the previous 3 months (B: 0.862; SD: 0.088; *p* <0.001), Child-Pugh score (B: 2.974; SD: 1.089; *p* = 0.012), whereas, on multivariate analysis, only alcohol use over the previous 3 months remained statistically significant (Table 3).

## Discussion

The number of studies investigating the validity of direct alcohol-use biomarkers in patients with ALD is limited.^4^ Yet, these are urgently needed to fully understand their utility for diagnosing different levels of alcohol use over specific diagnostic time windows, while taking into account possible confounding factors.^4^ In a well-characterized cohort of patients with ALD cirrhosis, we showed excellent diagnostic accuracies for uEtG, PEth, hEtG, and nEtG for the detection of any alcohol use, with complementary optimal detection windows of 3 days for uEtG, 3 weeks for PEth, and 3 months for hEtG and nEtG.Furthermore, uEtG, PEth, hEtG, and nEtG showed high sensitivity and NPV to exclude increased and excessive alcohol use, which is of interest for categorizing patients with steatotic liver disease. Together, these data provide a framework for interpreting and applying direct alcohol-use biomarkers in patients with liver disease, facilitating their clinical implementation.

The diagnostic accuracy of blood PEth in patients with liver disease has only been assessed in a few studies.^4^^,^^11^^,^^23^ These included patients with varying stages of liver disease and did not consistently use the Timeline Followback method as the gold standard for alcohol use. In our study, PEth showed excellent diagnostic accuracy for any alcohol use over the previous 3 weeks, thereby filling the detection window gap between uEtG and hEtG. PEth and uEtG showed slightly better diagnostic accuracies compared with the long-term biomarkers hEtG and nEtG. This could be explained by distorted growth patterns of scalp hair due to cirrhosis, leading to interpatient variability.^8^ Slower hair growth or a higher percentage of hair in the telogen phase might leave remnant hEtG in the proximal hair segment or lead to altered incorporation of newly formed EtG.^8^ Since hair can take up to 1–2 weeks to grow out of the scalp, there might be a gap in the detection window for the 2 most recent weeks before inclusion.^24^ Similarly, fingernails might also have differing lengths and different growth patterns within and between patients, possibly confounding test results,^8^ indicating that our pilot data on nEtG need further validation before clinical implementation. Finally, recall bias might have a greater impact over more extended time periods compared with shorter periods. Whereas the added value of hEtG lies in its long detection window of 3 months, an important advantage of PEth is the straightforward collection of blood samples.

Liver dysfunction can lead to decreased biomarker formation in diseased hepatocytes.^4^ By contrast, it has been hypothesized that, in patients with liver disease, nonoxidative pathways of ethanol metabolism might degrade a greater proportion of the ingested ethanol compared with oxidative pathways, leading to elevated levels of direct biomarkers.^25^ In our cohort, we observed a correlation between Child-Pugh scores and absolute biomarker levels of PEth, hEtG, and nEtG on univariate analysis. After accounting for alcohol use, this correlation was only observed for PEth. Yet, only 9.1% of the included patients had Child-Pugh C cirrhosis. Impaired kidney function (resulting from impaired or delayed urinary excretion) or the use of diuretics (urinary dilution) can affect urinary biomarker levels. Although we did not observe an impact of kidney dysfunction on biomarker levels, we found a negative correlation between absolute levels of uEtG and the use of loop diuretics. However, only 4.5% of the included patients had KDIGO stage 4 or 5. Therefore, uEtG results should still be interpreted with caution in patients with severe kidney dysfunction^26^^,^^27^ and those who take diuretics. Although we demonstrated excellent diagnostic accuracies of the respective alcohol-use biomarkers, further validation of these biomarkers in patients with advanced cirrhosis should be the focus of future studies. Furthermore, we found no effect of BMI, anemia, or sex on biomarker levels. We also did not observe important differences in diagnostic accuracy between sex-adjusted and non-sex-adjusted alcohol-use thresholds (Table S1).

Significant alcohol use determines the clinical course in patients with steatotic liver disease and metabolic dysfunction. Recently, to better define the natural history and facilitate biomarker and therapy development, the terms ‘MASLD’ and ‘MetALD’ in addition to ALD were introduced.^12^ These form a spectrum of steatotic liver disease in which patients are categorized based on the presence of features of metabolic dysfunction and their exact level of alcohol use. However, this categorization heavily relies on self-reporting, which is notoriously unreliable, at least in a subset of patients with steatotic liver disease. Recently, Staufer *et al.* showed that, in patients with presumed MASLD, >25% had moderate or excessive alcohol use, underscoring the need for objective tools to detect alcohol use.^2^ Therefore, we explored the diagnostic accuracy of direct alcohol-use biomarkers for increased or excessive use, in addition to any alcohol use.^12^ Interestingly, within our cohort, uEtG, Peth, and hEtG yielded a high sensitivity and NPV for both increased and excessive use, making them excellent and timely tests to rule out significant alcohol use in patients with (presumed) MASLD. We speculate that the observed lower specificity of direct alcohol-use biomarkers for increased and excessive alcohol use could be explained by several factors. First, there might be a longer half-life of the biomarker when larger amounts of alcohol are ingested. The larger amount of alcohol use could further reinforce possible differences in biomarker formation and excretion between patients, also when using the same amount of alcohol. High levels of uEtG could also indicate that patients had moderate amounts of alcohol during the hours immediately before sampling, which should be taken into account when assessing excessive use by uEtG.^28^ The lower specificity could also be explained by a proportion of patients who admit recent alcohol use, but under-report their exact level of use. Further validation studies are necessary to examine the diagnostic power of alcohol-use biomarkers and their appropriate cut-off levels to ‘rule-in’ patients with increased and excessive alcohol use. In light of steatotic liver disease, a single alcohol-use biomarker measurement does not provide information on the historical alcohol use of an individual patient. Alcohol use can change over time, whereas historical use co-determines the current liver phenotype of a patient. However, this challenge could be overcome by repetitive measurements per patient.

An unavoidable limitation in validation studies regarding alcohol-use biomarkers is the lack of a gold standard, which thus indicates the need for accurate alcohol-use biomarkers. Within this study, we attempted to minimize this bias by only including patients independently assessed for reliability by two physicians and by using the Alcohol Timeline Followback Method, which allowed us to accurately assess varying levels of alcohol use per specific detection window. This method provides quantitative information on a day-to-day basis, in contrast to the frequently used alcohol use disorder identification test (AUDIT) or modified AUDIT scores.^2^^,^^4^ Nevertheless, it has its inherent limitations, and future studies should ideally include prospective self-reporting of alcohol use. Despite their good to excellent diagnostic accuracy, uEtG, PEth, and hEtG all had a limited number of positive results in patients denying alcohol use. However, all patients with a negative alcohol-use history during the analyzed time frame and positive uEtG (n = 5) or PEth (n = 1) had alcohol use during the period before the detection windows of 3 days and 3 weeks.

## Conclusions

uEtG, Peth, and hEtG are complementary biomarkers that overcome their individual limitations. These markers allow us to obtain a reliable alcohol-use profile of patients with steatotic liver disease, in both clinical care and the context of pharmacological trials.

## Abbreviations

ALD, alcohol-related liver disease; ALT, alanine transaminase; AST, aspartate transferase; AUDIT, alcohol use disorder identification test; AUROC, area under the receiver operating characteristic curve; B, unstandardized coefficient; CDT, carbohydrate-deficient transferrin; CDT_(CZE)_, carbohydrate-deficient by capillary zone electrophoresis; CDT_(Nef)_, carbohydrate-deficient transferrin by nephelometric analysis; CZE, capillary zone electrophoresis; EGFR, estimated glomerular filtration rate; EtG, ethyl glucuronide; GC-MS/MS, gas chromatography-tandem mass spectrometry; GGT, gamma-glutamyl transferase; hEtG, hair ethyl glucuronide; IFCC, International Federation of Clinical Chemistry and Laboratory Medicine; INR, International Normalized Ratio; KDIGO, Kidney Disease Improving Global Outcomes; LC-MS/MS, liquid chromatography-tandem mass spectrometry; MASLD, metabolic dysfunction-associated liver disease; MCV, mean corpuscular volume; MELD, Model for End-Stage Liver Disease; MetALD, metabolic dysfunction and alcohol associated/related liver disease; Nef, nephelometry; nEtG, nail ethyl glucuronide; NPV, negative predictive value; PEth, phosphatidylethanol; PPV, positive predictive value; ROC, receiver operating characteristic curve; STARD, Standards for Reporting of Diagnostic Accuracy Studies; uEtG, urinary ethyl glucuronide; uEtS, urinary ethyl sulfate.

## Financial support

The study was supported by the 10.13039/501100008359Maag Lever Darm Stichting (Dutch Digestive Foundation)–Grant number D 18-19.

## Authors’ contributions

BV, JV: participated in the conceptualization, research design, data analysis, and drafting of the manuscript and are responsible for the accuracy and integrity of all aspects of the work. JV: acquired project funding and supervised the project. CD, HN, AN: performed nEtG, hEtG, and PEth analysis and critically revised the manuscript. NE: performed uEtG and ethyl sulfate analysis and critically revised the manuscript. DB: performed CDT analysis and critically revised the manuscript. AM, LM, HM, TG, MK, SM, FN: critically revised the manuscript. All authors contributed to draft finalization and approved the final version of the manuscript.

## Data availability statement

The data supporting this study's findings are available from the corresponding author upon request.

## Conflicts of interest

The authors declare no conflicts of interest.

Please refer to the accompanying ICMJE disclosure forms for further details.
