# Supplementary material for: Phosphatidylethanol and ethyl glucuronide to categorize alcohol consumption in alcohol-related cirrhosis
Source: JHEP Rep. 2025 Apr 24;7(8):101433. doi: 10.1016/j.jhepr.2025.101433 (PMC12269596; doi:10.1016/j.jhepr.2025.101433)

# Phosphatidylethanol and ethyl glucuronide to categorize alcohol consumption in alcohol-related cirrhosis

Benedict T.K. Vanlerberghe, Catalina Dumitrascu, Nele Van den Eede, Hugo Neels, Hannah van Malenstein, Tom J.G. Gevers, Matthijs Kramer, Lukas Van Melkebeke, Ad A.M. Masclee, Douwe de Boer, Schalk van der Merwe, Frederik Nevens, Alexander L.N. van Nuijs, Jef Verbeek

Table of contents

Table S1…………………………………………………………………………………2

Table S2…………………………………………………………………………………3

Table S3…………………………………………………………………………………4

Table S4…………………………………………………………………………………5

Table S5…………………………………………………………………………………6

Table S6…………………………………………………………………………………7

Fig. S1…………………………………………………………………………………...9

Table S1: diagnostic accuracy of PEth, $CDT_{(Nef)}$, $CDT_{(CZE)}$, hEtG, and nEtG to detect increased or excessive alcohol use with increased and excessive use not corrected for sex

| *Alcohol use biomarker* | *AUROC (95%CI)* | *Sensitivity (%)* | *Specificity (%)* | *PPV (%)* | *NPV (%)* | *Youden-Index* |
|---|---|---|---|---|---|---|
| **Short-term alcohol use biomarkers** | | | | | | |
| Short-term alcohol use biomarkers reflecting 3 day increased alcohol use | | | | | | |
| uEtG (≥ 9 mg/g creat) | 0.982 (0.956 – 1.000) | 81.8 | 97.7 | 81.8 | 97.7 | 0.795 |
| Short-term alcohol use biomarkers reflecting 3 day excessive alcohol use | | | | | | |
| uEtG (≥ 9 mg/g creat) | 0.983 (0.956 – 1.000) | 100.0 | 93.5 | 45.5 | 100.0 | 0.935 |
| **Mid-term alcohol use biomarkers** | | | | | | |
| Mid-term alcohol use biomarkers reflecting 3 week increased alcohol use | | | | | | |
| PEth (≥ 200 ng/mL) | 0.926 (0.878-0.975) | 75.0 | 87.7 | 64.3 | 92.2 | 0.627 |
| $CDT_{(Nef)}$ (> 1.7%) | 0.737 (0.603-0.872) | 41.7 | 91.7 | 58.8 | 84.6 | 0.334 |
| $CDT_{(CZE)}$ (> 1.7%) | 0.807 (0.675-0.938) | 42.1 | 93.4 | 61.5 | 86.6 | 0.355 |
| Mid-term alcohol use biomarkers reflecting 3 week excessive alcohol use | | | | | | |
| PEth (≥ 200 ng/mL) | 0.921 (0.861-0.980) | 83.3 | 80.6 | 35.7 | 97.4 | 0.639 |
| $CDT_{(Nef)}$ (> 1.7%) | 0.665 (0.445-0.885) | 50.0 | 88.5 | 35.3 | 93.4 | 0.385 |
| $CDT_{(CZE)}$ (> 1.7%) | 0.745 (0.540-0.949) | 33.3 | 88.4 | 23.1 | 92.7 | 0.217 |
| **Long-term alcohol use biomarkers** | | | | | | |
| Long-term alcohol use biomarkers reflecting 3 month increased alcohol use | | | | | | |
| hEtG (≥ 30 pg/mg) | 0.898 (0.816-0.980) | 88.5 | 87.3 | 71.9 | 95.4 | 0.758 |
| nEtG (≥ 123 pg/mg) | 0.940 (0.884-0.995) | 84.6 | 90.2 | 81.5 | 92.0 | 0.748 |
| Long-term alcohol use biomarkers reflecting 3 month excessive alcohol use | | | | | | |
| hEtG (≥ 30 pg/mg) | 0.945 (0.899-0.991) | 100.0 | 79.3 | 46.9 | 100.0 | 0.793 |
| nEtG (≥ 123 pg/mg) | 0.946 (0.889-0.994) | 94.4 | 83.1 | 63.0 | 98.0 | 0.775 |

Legend: Data are reported as AUROC and 95%CI, sensitivity, specificity, positive predictive value, negative predictive value, and Youden Index as appropriate.

Abbreviations: AUROC: area under the receiver operating characteristic curve; CI: confidence interval; PPV: positive predictive value; NPV: negative predictive value; PEth: phosphatidylethanol; $CDT_{(Nef)}$: carbohydrate-deficient transferrin by nephelometric analysis; $CDT_{(CZE)}$: carbohydrate-deficient transferrin by capillary zone electrophoresis.

Table S2: diagnostic accuracy of uEtG and uEtS to detect any alcohol use over the last three days not corrected for urinary creatinine

| *Alcohol use biomarker* | *AUROC (95%CI)* | *Sensitivity (%)* | *Specificity (%)* | *PPV (%)* | *NPV (%)* | *Youden-Index* |
|---|---|---|---|---|---|---|
| Short-term alcohol use biomarkers reflecting 3 day alcohol use | | | | | | |
| uEtG (≥ 0.1 mg/L) | 0.989 (0.972 – 1.000) | 100.0 | 86.8 | 68.8 | 100.0 | 0.868 |
| uEtS (≥ 0.1 mg/L) | 0.956 (0.899 – 1.000) | 95.5 | 85.5 | 65.6 | 98.5 | 0.810 |

Legend: Data are reported as AUROC and 95%CI, sensitivity, specificity, positive predictive value, negative predictive value, and Youden Index as appropriate.

Abbreviations: AUROC: area under the receiver operating characteristic curve; CI: confidence interval; PPV: positive predictive value; NPV: negative predictive value; uEtG: urinary ethyl glucuronide; create: creatinine; uEtS: urinary ethyl sulfate.

Table S3: diagnostic accuracy of MCV and liver function tests in detecting any alcohol use over 3 days, 3 weeks and 3 months.

| *Liver function test* | *AUROC (95%CI)* | *Sensitivity (%)* | *Specificity (%)* | *PPV (%)* | *NPV (%)* | *Youden-Index* |
|---|---|---|---|---|---|---|
| | Liver tests reflecting 3 day alcohol use | | | | | |
| MCV | 0.606 (0.485-0.727) | 63.0 | 53.0 | 30.4 | 81.5 | 0.160 |
| yGT | 0.817 (0.728-0.907) | 92.6 | 41.0 | 33.8 | 94.4 | 0.336 |
| AST | 0.731 (0.618-0.843) | 81.5 | 53.0 | 36.1 | 89.8 | 0.345 |
| AST/ALT* | 0.698 (0.577-0.820) | 70.4 | 59.0 | 35.8 | 86.0 | 0.294 |
| | Liver tests reflecting 3 week alcohol use | | | | | |
| MCV | 0.713 (0.541-0.885) | 65.1 | 58.2 | 50.0 | 72.2 | 0.232 |
| yGT | 0.826 (0.744-0.908) | 86.0 | 44.8 | 50.0 | 83.3 | 0.308 |
| ASAT | 0.732 (0.629-0.836) | 74.4 | 56.7 | 52.5 | 77.6 | 0.311 |
| AST/ALT* | 0.615 (0.499-0.730) | 62.8 | 61.2 | 50.9 | 71.9 | 0.240 |
| | Liver tests biomarkers reflecting 3 month alcohol use | | | | | |
| MCV | 0.639 (0.535-0.743) | 60.4 | 56.5 | 51.8 | 64.8 | 0.169 |
| yGT | 0.810 (0.727-0.893) | 83.3 | 45.2 | 54.1 | 77.8 | 0.285 |
| ASAT | 0.717 (0.616-0.817) | 70.8 | 56.5 | 55.7 | 71.4 | 0.273 |
| AST/ALT* | 0.618 (0.506-0.730) | 64.6 | 64.5 | 58.5 | 70.2 | 0.291 |

Legend: Data are reported as AUROC and 95%CI, sensitivity, specificity, positive predictive value, negative predictive value, and Youden Index as appropriate.

Abbreviations: AUROC: area under the receiver operating characteristic curve; CI: confidence interval; PPV: positive predictive value; NPV: negative predictive value; MCV: mean corpuscular volume; yGT: Gamma-glutamyltransferase; AST: aspartate transferase; ALT: alanine transaminase; AST/ALT ratio over 1.5.

Table S4: diagnostic accuracy of uEtG and uEtS to detect any alcohol use over 1- to 7-day detection windows

| *Alcohol use biomarker* | *AUROC (95%CI)* | *Sensitivity (%)* | *Specificity (%)* | *PPV (%)* | *NPV (%)* | *Youden-Index* |
|---|---|---|---|---|---|---|
| Short-term alcohol use biomarkers reflecting 1 day alcohol use (with 13 having any alcohol use) | | | | | | |
| uEtG (≥ 0.121 mg/g creat) | 0.984 (0.963-1.000) | 100 | 84.5 | 48.1 | 100 | 0.845 |
| uEtS (≥ 0.108 mg/g creat) | 0.981 (0.954-1.000) | 100 | 82.4 | 46.4 | 100 | 0.824 |
| Short-term alcohol use biomarkers reflecting 2 day alcohol use (with 17 having any alcohol use) | | | | | | |
| uEtG (≥ 0.121 mg/g creat) | 0.983 (0.963-1.000) | 100 | 87.6 | 63.0 | 100 | 0.876 |
| uEtS (≥ 0.108 mg/g creat) | 0.980 (0.954-1.006) | 94.1 | 85.2 | 57.1 | 98.6 | 0.793 |
| Short-term alcohol use biomarkers reflecting 3 day alcohol use (with 22 having any alcohol use) | | | | | | |
| uEtG (≥ 0.121 mg/g creat) | 0.990 (0.975-1.000) | 100 | 93.4 | 81.5 | 100 | 0.943 |
| uEtS (≥ 0.108 mg/g creat) | 0.948 (0.889-1.000) | 86.4 | 88.2 | 67.9 | 95.7 | 0.746 |
| Short-term alcohol use biomarkers reflecting 4 day alcohol use (with 23 having any alcohol use) | | | | | | |
| uEtG (≥ 0.121 mg/g creat) | 0.965 (0.911-1.000) | 95.7 | 93.3 | 81.5 | 98.6 | 0.890 |
| uEtS (≥ 0.108 mg/g creat) | 0.925 (0.850-1.000) | 82.6 | 88.0 | 67.9 | 94.3 | 0.706 |
| Short-term alcohol use biomarkers reflecting 5 day ( = 6 day) alcohol use (with 24 having any alcohol use) | | | | | | |
| uEtG (≥ 0.121 mg/g creat) | 0.961 (0.909-1.010) | 91.7 | 93.2 | 81.5 | 97.2 | 0.849 |
| uEtS (≥ 0.108 mg/g creat) | 0.903 (0.818-0.989) | 79.2 | 87.8 | 67.9 | 92.9 | 0.670 |
| Short-term alcohol use biomarkers reflecting 7 day alcohol use (with 27 having any alcohol use) | | | | | | |
| uEtG (≥ 0.121 mg/g creat) | 0.947 (0.884-1.000) | 88.9 | 95.8 | 88.9 | 95.8 | 0.847 |
| uEtS (≥ 0.108 mg/g creat) | 0.869 (0.775-0.964) | 74.1 | 88.7 | 71.4 | 90 | 0.628 |

Legend: Data are reported as AUROC and 95%CI, sensitivity, specificity, positive predictive value, negative predictive value, and Youden Index as appropriate.

Abbreviations: AUROC: area under the receiver operating characteristic curve; CI: confidence interval; PPV: positive predictive value; NPV: negative predictive value; uEtG: urinary ethyl glucuronide; creat: creatinine; uEtS: urinary ethyl sulfate.

Table S5: diagnostic accuracy of PEth, $CDT_{(Nef)}$ and $CDT_{(CZE)}$ to detect any alcohol use over 1- to 5-week detection windows

| *Alcohol use biomarker* | *AUROC (95%CI)* | *Sensitivity (%)* | *Specificity (%)* | *PPV (%)* | *NPV (%)* | *Youden-Index* |
|---|---|---|---|---|---|---|
| Mid-term alcohol use biomarkers reflecting 7 day alcohol use (with 31 having any alcohol use) | | | | | | |
| PEth (≥ 20 ng/mL) | 0.966 (0.934-0.999) | 100 | 86.5 | 75.6 | 100 | 0.865 |
| $CDT_{(Nef)}$ (> 1.7%) | 0.718 (0.610-0.826) | 31.3 | 90.8 | 58.8 | 75.80 | 0.221 |
| $CDT_{(CZE)}$ (> 1.7%) | 0.807 (0.691-0.923) | 35.7 | 95.5 | 76.9 | 78.0 | 0.312 |
| Mid-term alcohol use biomarkers reflecting 14 day alcohol use (with 37 having any alcohol use) | | | | | | |
| PEth (≥ 20 ng/mL) | 0.963 (0.923-1.000) | 97.3 | 92.6 | 87.8 | 98.4 | 0.899 |
| $CDT_{(Nef)}$ (> 1.7%) | 0.684 (0.572-0.797) | 28.9 | 91.4 | 64.7 | 70.3 | 0.203 |
| $CDT_{(CZE)}$ (> 1.7%) | 0.772 (0.655-0.888) | 32.3 | 95.3 | 76.9 | 74.4 | 0.265 |
| Mid-term alcohol use biomarkers reflecting 21 day alcohol use (with 42 having any alcohol use) | | | | | | |
| PEth (≥ 20 ng/mL) | 0.986 (0.958-1.000) | 95.2 | 98.4 | 97.6 | 96.9 | 0.936 |
| $CDT_{(Nef)}$ (> 1.7%) | 0.721 (0.617-0.825) | 30.2 | 93.8 | 76.5 | 67.0 | 0.240 |
| $CDT_{(CZE)}$ (> 1.7%) | 0.744 (0.630-0.859) | 30.6 | 96.6 | 84.6 | 69.5 | 0.272 |
| Mid-term alcohol use biomarkers reflecting 28 day alcohol use (with 43 having any alcohol use) | | | | | | |
| PEth (≥ 20 ng/mL) | 0.974 (0.936-1.000) | 93.0 | 98.4 | 97.6 | 95.3 | 0.914 |
| $CDT_{(Nef)}$ (> 1.7%) | 0.734 (0.632-0.836) | 31.8 | 95.3 | 82.4 | 67.0 | 0.271 |
| $CDT_{(CZE)}$ (> 1.7%) | 0.725 (0.608-0.842) | 0.297 | 96.6 | 84.6 | 68.3 | 0.263 |
| Mid-term alcohol use biomarkers reflecting 35 day alcohol use (with 45 having any alcohol use) | | | | | | |
| PEth (≥ 20 ng/mL) | 0.965 (0.921-1.000) | 91.1 | 100 | 100 | 93.8 | 0.911 |
| $CDT_{(Nef)}$ (> 1.7%) | 0.714 (0.610-0.818) | 30.4 | 95.2 | 82.4 | 64.8 | 0.256 |
| $CDT_{(CZE)}$ (> 1.7%) | 0.714 (0.596-0.832) | 28.2 | 96.4 | 84.6 | 65.9 | 0.246 |

Legend: Data are reported as AUROC and 95%CI, sensitivity, specificity, positive predictive value, negative predictive value, and Youden Index as appropriate.

Abbreviations: AUROC: area under the receiver operating characteristic curve; CI: confidence interval; PPV: positive predictive value; NPV: negative predictive value; PEth: phosphatidylethanol; $CDT_{(Nef)}$: carbohydrate-deficient transferrin by nephelometric analysis; $CDT_{(CZE)}$: carbohydrate-deficient transferrin by capillary zone electrophoresis.

Table S6: Comparison of predefined cut-offs of alcohol use biomarkers to the optimal cut-off of alcohol use biomarkers based on the highest Youden Index.

| *Predefined cut-off* | *Optimal cut-off based on highest Youden Index* | *SE / SP optimal cut-off (predefined cut-off)* | *FN optimal cutoff (predefined cut-off)* | *FP optimal cutoff (predefined cut-off)* | *N of patients correctly diagnosed more by the optimal cut-off* |
|---|---|---|---|---|---|
| **Short-term alcohol use biomarkers** | | | | | |
| Short-term alcohol use biomarkers reflecting 3 day any alcohol use | | | | | |
| uEtG (≥ 0.121 mg/g creat) | uEtG (≥ 0.126 mg/g creat) | SE 100.0 (100.0)<br>SP 93.4 (93.4) | 0 (0) | 5 (5) | 0 |
| uEtS (≥ 0.108 mg/g creat) | uEtS (≥ 0.033 mg/g creat) | SE 95.5 (86.4)<br>SP 85.5 (88.2) | 1 (3) | 11 (9) | 0 |
| Short-term alcohol use biomarkers reflecting 3 day increased alcohol use | | | | | |
| uEtG (≥ 9 mg/g creat) | uEtG (≥ 2.48 mg/g creat) | SE 91.7 (75.0)<br>SP 96.5 (97.7) | 1 (3) | 3 (2) | 1 |
| Short-term alcohol use biomarkers reflecting 3 day excessive alcohol use | | | | | |
| uEtG (≥ 9 mg/g creat) | uEtG (≥ 14.9 mg/g creat) | SP 100 (100.0)<br>SP 94.6 (93.5) | 0 (0) | 5 (6) | 1 |
| **Mid-term alcohol use biomarkers** | | | | | |
| Mid-term alcohol use biomarkers reflecting 3 week any alcohol use | | | | | |
| PEth (≥ 20 ng/mL) | PEth (≥ 12.9 ng/mL) | SP 97.6 (95.2)<br>SP 96.8 (98.4) | 1 (2) | 2 (1) | 0 |
| Mid-term alcohol use biomarkers reflecting 3 week increased alcohol use | | | | | |
| PEth (≥ 200 ng/mL) | PEth (≥ 33.4 ng/mL) | SP 96.2 (76.9)<br>SP 83.5 (89.9) | 1 (6) | 13 (8) | 0 |
| Mid-term alcohol use biomarkers reflecting 3 week excessive alcohol use | | | | | |
| PEth (≥200 ng/mL) | PEth (≥ 145.8 ng/mL) | SP 91.7 (83.3)<br>SP 80.6 (80.6) | 1 (2) | 18 (18) | 1 |
| **Long-term alcohol use biomarkers** | | | | | |
| Long-term alcohol use biomarkers reflecting 3 month any alcohol use | | | | | |
| hEtG (≥ 5 pg/mg) | hEtG (≥ 11.5 pg/mg) | SP 84.6 (89.7) | 6 (4) | 4 (8) | 2 |

| | | | | | |
|---|---|---|---|---|---|
| | | SP 91.5 (86.2) | | | |
| nEtG (≥ 59 pg/mg) | nEtG (≥ 46.4 pg/mg) | SP 86.8 (78.9)<br>SP 94.9 (97.4) | 5 (8) | 2 (1) | 2 |
| Long-term alcohol use biomarkers reflecting 3 month increased alcohol use | | | | | |
| hEtG (≥ 30 pg/mg) | hEtG (≥ 29.7 pg/mg) | SP 89.3 (89.3)<br>SP 89.9 (89.9) | 3 (3) | 7 (7) | 0 |
| nEtG (≥ 123 pg/mg) | nEtG (≥ 74.9 pg/mg) | SP 92.6 (85.2)<br>SP 90.0 (92.0) | 2 (4) | 5 (4) | 1 |
| Long-term alcohol use biomarkers reflecting 3 month excessive alcohol use | | | | | |
| hEtG (≥ 30 pg/mg) | hEtG (≥ 29.7 pg/mg) | SP 100.0 (100.0)<br>SP 79.3 (79.3) | 0 (0) | 17 (17) | 0 |
| nEtG (≥ 123 pg/mg) | nEtG (≥ 159.2 pg/mg) | SP 94.4 (94.4)<br>SP 89.8 (83.1) | 1 (1) | 6 (10) | 4 |

Legend: Data are reported as sensitivity, specificity, positive predictive value, negative predictive value as appropriate.

Abbreviations: AUROC: SE: sensitivity; SP: specificity; FN: false negative; FP: false positive; N: number; uEtG: urinary ethyl glucuronide; creat: creatinine; uEtS: urinary ethyl sulfate, PEth: phosphatidylethanol; hEtG: scalp hair ethyl glucuronide; nEtG: fingernail ethyl glucuronide.

Fig. S1: Flow diagram for patients included in the overall analysis

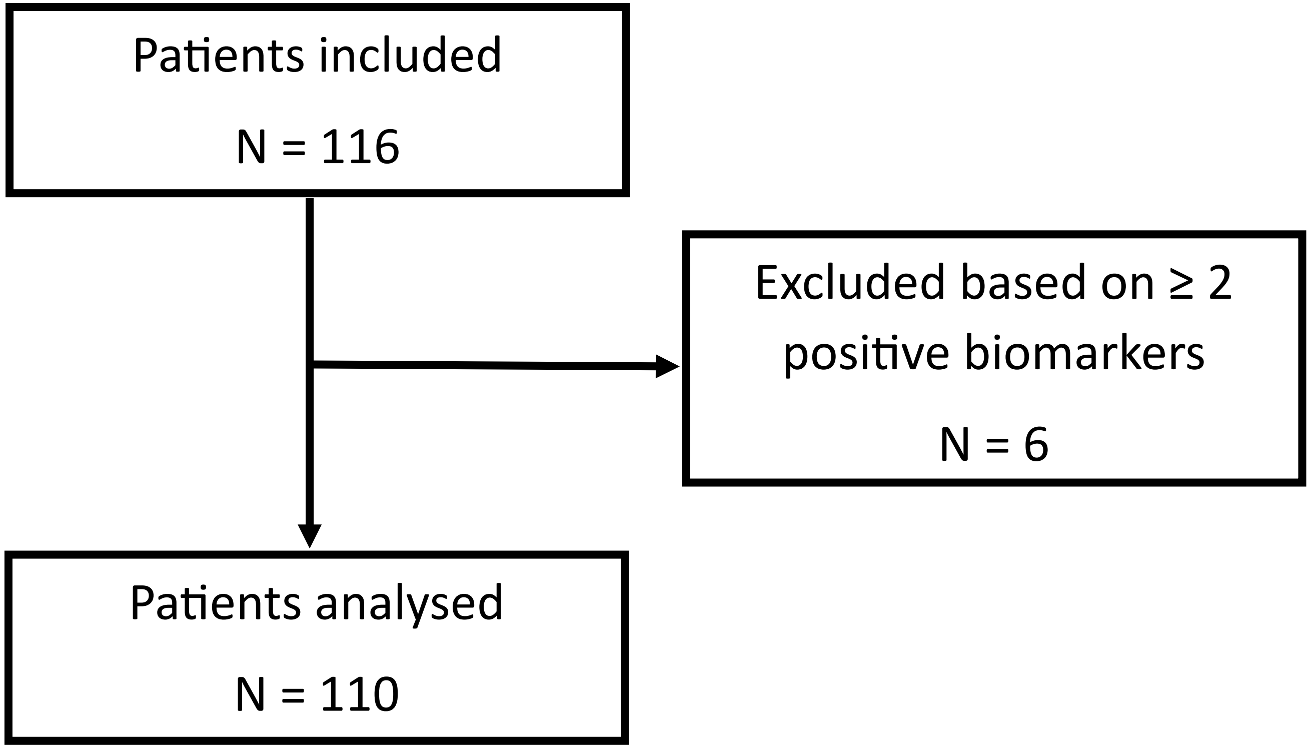

Supplement: Multimedia component 1 [file mmc1.pdf]
